# Daldiniaeschsone A, a Rare Tricyclic Polyketide Having a Chromone Unit Fused to a *δ*-Lactone and Its Symmetrical Biphenyl Dimer, Daldiniaeschsone B, from an Endophytic Fungus *Daldinia eschscholtzii* SDBR-CMUNKC745

**DOI:** 10.3390/jof7050358

**Published:** 2021-05-02

**Authors:** Natnicha Wutthiwong, Virayu Suthiphasilp, Aknarin Pintatum, Nakarin Suwannarach, Jaturong Kumla, Saisamorn Lumyong, Tharakorn Maneerat, Rawiwan Charoensup, Sarot Cheenpracha, Thunwadee Limtharakul, Stephen G. Pyne, Surat Laphookhieo

**Affiliations:** 1 Department of Biology, Faculty of Science, Chiang Mai University, Chiang Mai 50200, Thailand; natnicha.mrp@hotmail.com (N.W.); suwan.462@gmail.com (N.S.); Jaturong_yai@hotmail.com (J.K.); 2 Research Center of Microbial Diversity and Sustainable Utilization, Chiang Mai University, Chiang Mai 50200, Thailand; 3 Center of Chemical Innovation for Sustainability (CIS), School of Science, Mae Fah Luang University, Chiang Rai 57100, Thailand; virayu.suthiphasilp@gmail.com (V.S.); p.aknarin@gmail.com (A.P.); wisanu.man@mfu.ac.th (T.M.); 4 Academy of Science, The Royal Society of Thailand, Bangkok 10300, Thailand; 5 Medicinal Plants Innovation Center of Mae Fah Luang University, Chiang Rai 57100, Thailand; rawiwan.cha@mfu.ac.th; 6 School of Integrative Medicine, Mae Fah Luang University, Chiang Rai 57100, Thailand; 7 School of Science, University of Phayao, Phayao 56000, Thailand; cheenpracha@gmail.com; 8 Department of Chemistry, Faculty of Science and Research Center on Chemistry for Development of Health Promoting Products from Northern Resources, Chiang Mai University, Chiang Mai 50200, Thailand; othunwadee@gmail.com; 9 School of Chemistry and Molecular Bioscience, University of Wollongong, Wollongong, NSW 2522, Australia; spyne@uow.edu.au

**Keywords:** *Daldinia eschscholtzii* SDBR-CMUNKC745, tricyclic polyketide, biphenyl dimer, chromone, *α*-glucosidase inhibitory activity

## Abstract

Daldiniaeschsone A (**1**), a rare tricyclic polyketide having a chromone unit fused to a *δ*-lactone and its symmetrical 6,6′-biphenyl dimer, daldiniaeschsone B (**2**), together with three known compounds (**3**−**5**), were isolated from a plant-derived endophytic fungus, *Daldinia eschscholtzii* SDBR-CMUNKC745. Their structures were elucidated by extensive 1D and 2D NMR spectroscopic data and HRESIMS. All compounds showed *α*-glucosidase inhibitory activity with IC_50_ values ranging from 0.16−0.85 mM and compound **1** was the best *α*-glucosidase inhibitory activity (IC_50_ = 0.16 mM).

## 1. Introduction

Natural products from fungi have been observed to inhibit or kill a wide variety of harmful microorganisms, including, but not limited to, phytopathogens and bacteria, fungi, viruses, and protozoans that affect humans and animals [1,2,3]. Similar to plants, many compounds isolated from microorganisms have become well-known drugs such as penicillin (*Penicillium notatum*), vancomycin (*Amycolatopsis orientalis*), and erythromycin (*Saccharopolyspora erythraea*) [4]. Due to the short period to scale up and the diversity production of secondary metabolites, fungi have become the new resource for natural products. The genus *Daldinia* belonging to the Xylariaceae is a genus of fungi that produces polyketide compounds with diverse structures and impressive biological activities, including immunosuppressive, cytotoxic, antiangiogenesis, anti-inflammatory, and antibacterial activities [1,2,5,6,7,8]. The two most investigated *Daldinia* species are *D. eschscholtzii* and *D. concentrica* which have produced many new compounds having remarkable biological activities including *α*-glucosidase inhibitory, antiangiogenesis, and anti-HIV activities [1,2,9,10].

*Daldinia eschscholtzii* is a wood-inhabiting and decaying endophytic fungus distributed in warm tropical and subtropical areas [11]. Previous chemical constituent investigation of *D. eschscholtzii* resulted in the discovery of various types of secondary metabolites, including chromones, lactones, phenolics, and alkaloids [1,12,13,14,15,16,17] Many of these isolated compounds have demonstrated a wide range of bioactivities, such as *α*-glucosidase inhibition [1], immunosuppression [12], antimicrobial [6,13,14,15], stem cell differentiation induction [16] and anti-inflammatory [17] activities. In the course of our ongoing search for new compounds and their biological activities from natural resources, the investigation of the broth extract of *D. eschscholtzii* SDBR-CMUNKC745 resulted in the isolation of two new chromone derivatives and three known compounds. In this study, we report the isolation and structure elucidation of two new compounds (**1** and **2**). The *α*-glucosidase and NO production inhibition activities of all compounds are also reported.

## 2. Materials and Methods

### 2.1. General Experimental Procedures

The optical rotations were measured with a Jasco P-1010 polarimeter. The UV spectra were recorded with a PerkinElmer UV–Vis spectrophotometer. Infrared (IR) spectra were recorded using a PerkinElmer Frontier Optica FT–IR spectrometer. The 1D and 2D NMR spectra recorded using a 500 MHz Bruker AV-500 spectrometer. Chemical shifts are reported in parts per million (*δ*) and coupling constants (*J*) are expressed in hertz. The HRESIMS were carried out on a QTOF 6540 UHD mass spectrometer (Agilent Technologies, Santa Clara, CA, USA). Chiral HPLC was performed on a CHIRALCEL OD-H column of 4.6*ϕ*—250 mm and attached to Agilent Technologies 1260 Infinity II. Silica gel G60 (60–200 µm, SiliCycle^®^ Inc., Québec, QC G1P 4S6, Canada) was used to perform column chromatography (CC). Precoated plates of silica gel (60F254, Merck, Kenilworth, NJ, USA) were used for analytical purposes.

### 2.2. Fungal Material

**Figure 1:**
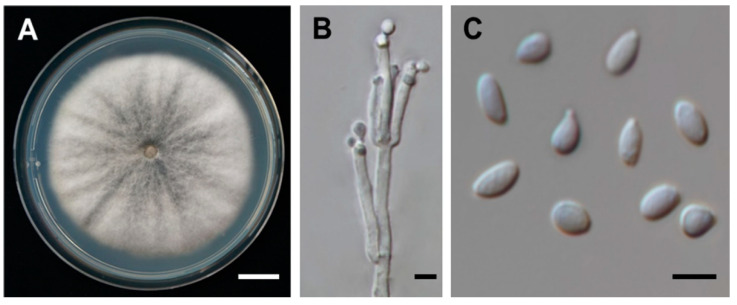
*Daldinia eschscholtzii* SDBR-CMUNKC745. (**A**) Colony on PDA at 30 °C for one week. (**B**) Conidiophores and conidiogeneous cells. (**C**) Conidia. Scale bars: (**A**) = 10 mm, (**B**) = 1 μm, (**C**) = 5 μm.

**Figure 2:**
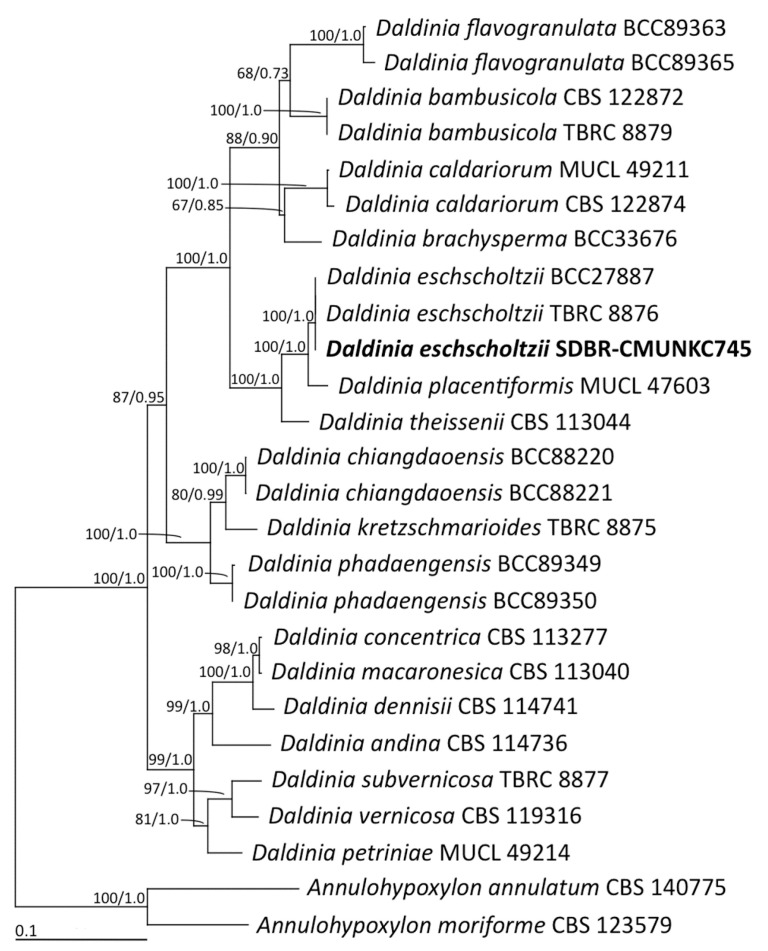
Phylogram derived from maximum likelihood analysis of the combined ITS, LSU, RBP2, and TUB genes of 26 fungal taxa. Sequences of *Annulohypoxylon annulatum* and *A. moriforme* were used as outgroup. The numbers above branches represent maximum likelihood bootstrap percentages (left) and Bayesian posterior probabilities (right). Only bootstrap values ≥ 50% and Bayesian posterior probabilities ≥ 0.70 are shown. The scale bar represents ten substitutions per nucleotide position. Sequences of fungal species obtained in this study are in bold.

The fungus strain SDBR-CMUNKC745 was isolated from *Cinnamomum bejolghota*, collected from Chiang Mai, Thailand, in October 2015. This strain was deposited in the Culture Collection of Sustainable Development of Biological Resources (SDBR) Laboratory, Faculty of Science, Chiang Mai University, Thailand. Fungal colonies on potato dextrose agar grew to 80−85 mm at 30 °C in darkness after one week. Colonies were white to pale grey, and the reverse side was whitish-grey and black in the centre (Figure 1A). Sporulation was observed within three weeks. Conidiophores were 0.8–2 μm long × 0.7–1.5 μm wide, hyaline, mononematous or synnematous, nodulisporium-like branching pattern with dichotomous or trichotomous branched (Figure 1B). Conidiogeneous cells were 2.8–3.1 × 2.5–2.9 μm, cylindrical, hyaline, and smooth. Conidia were 4.8–6.4 × 2–3.8 μm, holoblastic, hyaline, ellipsoid to obovoid, aseptate, and smooth to finely roughened with flattened base (Figure 1C). The internal transcribed spacer (ITS) and large subunit (LSU) region of ribosomal DNA, RNA polymerase II second largest subunit (RPB2), and β-tubulin (TUB) genes of this strain were deposited in GenBank under the accession number MW1937612 MW193761, MW202236, and MW202237, respectively. The phylogenetic tree from the combined data set of ITS, LSU, RBP2, and TUB indicated that strain SDBR-CMUNKC745 clustered with *D. eschscholtzii* with 100% bootstrap and 1.0 posterior probability supports (Figure 2). Moreover, each gene sequence of this strain revealed a similarity greater than 99% to the *D. eschscholtzii* MUCL 45435.

### 2.3. Fermentation, Extraction, and Isolation of Secondary Metabolites 

*Daldinia eschscholtzii* SDBR-CMUNKC745 from stock culture was cultivated on potato dextrose agar (Difco™, Becton, Dickinson and Company, Franklin Lakes, NJ, USA) at 30 °C for five days. Then, five fungal mycelial plugs (5 mm in diameter) were inoculated into 500 mL Erlenmeyer flask containing 150 mL of Czapek Dox broth (CDB; 30 g/L sucrose, 2 g/L NaNO_2_, 1 g/L K_2_HPO_4_, 0.5 g/L KCl, 0.5 g/L MgSO_4_, and 0.01 g/L Fe_2_SO_4_, pH 8). Cultivation was performed in the dark at 30 °C with shaking at 100 rpm on a reciprocal shaker. After 10 days of incubation, the cultures were filtrated by Whatman’s No. 1 paper to harvest the supernatant. After that, the culture filtrate of endophytic fungus was extracted twice with an equal volume of EtOAc (1:1 *v/v*). The upper solvent phase was collected and evaporated using a rotary evaporator. The crude extract was stored at −20 °C for further experiments.

After removing the solvent under reduced pressure, the culture medium was extracted with EtOAc to provide a crude extract (4.4 g), which was subjected to silica gel CC (60–200 µm, 8 × 70 cm) using a gradient of EtOAc-hexanes (0.5:4.5, 1:4, 1.5:3.5, 2:3, 2.5:2.5, 3:1.5, 3.5:1.5, 4:1, 4.5:0.5, 0.5:4.5, and 5:0, *v/v*, 200 mL each) to afford five fractions (F1–F5). Fraction F2 (1.96 g) was further fractionated by silica gel CC (60–200 µm, 6 × 70 cm) using EtOAc-hexanes (1:4, *v/v*, 2500 mL) to give nine subfractions (F2A–F2J). Compound **2** (8.0 mg) was obtained from subfraction F2D (80.6 mg) by silica gel CC (60–200 µm, 3 × 30 cm) using EtOAc-CH_2_Cl_2_ (1:50, *v/v*, 1000 mL), while compound **1** (4.4 mg) was isolated from subfraction F2I (132.8 mg) by silica gel CC (60–200 µm, 3 × 30 cm) using EtOAc-CH_2_Cl_2_ (1:20, *v/v*, 1000 mL). Fraction F3 (390.0 mg) was further fractionated by silica gel CC (60–200 µm, 4× 50 cm) using EtOAc-hexanes (1:4, *v/v*, 1500 mL) to afford 10 subfractions (F3A–F3J). Subfraction F3A (75.8 mg) was further purified by silica gel CC (60–200 µm, 3 × 30 cm) using EtOAc-hexanes (1:4, *v/v*, 1000 mL) to give compound **3** (8.4 mg). Compound **4** (10.1 mg) was obtained from subfraction F3H (19.0 mg), while compound **5** (3.7 mg) was afforded from subfraction F3J (86.0 mg) by silica gel CC (60–200 µm, 2 × 30 and 3 × 30 cm, respectively) using EtOAc-hexanes (1:4, *v/v*, 500 mL) and EtOAc-CH_2_Cl_2_ (1:20, *v/v*, 1000 mL), respectively (Supplementary Materials, Figure S22).

Daldiniaeschsone A (**1**): colourless amorphous solid; [α]^21^_D_ −1.2 (*c* 1, MeOH); UV (MeOH) *λ*_max_ (log *ε*) 209 (3.44), 272 (3.26), 354 (2.74) nm; IR (neat) *ν*_max_ 3590, 2960, 1788, 1760, 1651, 1628, 1356, 1224, 1158, 1054, 735 cm^−1^; ^1^H and ^13^C NMR data, see Table 1; HRESITOFMS *m/z* 321.0972 [M + H]^+^ (calcd for 321.0969, C_16_H_17_O_7_).

Daldiniaeschsone B (**2**): yellow amorphous solid; [α]^21^_D_ −3.9 (*c* 1, MeOH); UV (MeOH) *λ*_max_ (log *ε*) 204 (3.79), 261 (2.78), 368 (3.31) nm; ECD (MeOH, *c* 0.9 × 10^−4^) *λ*_max_ (Δ*ε*) 317 (+5.26), 273 (+5.40), 225 (−6.10) nm; IR (neat) *ν*_max_ 3594, 2922, 1790, 1760, 1643, 1628, 1432, 1207, 1062, 734 cm^−1^; ^1^H and ^13^C NMR data, see Table 1; HRESITOFMS *m/z* 661.1504 [M + Na]^+^ (calcd for 661.1528, C_32_H_30_NaO_14_).

### 2.4. Bioassays

#### 2.4.1. α-Glucosidase Inhibitory Activity

A colorimetric *α*-glucosidase assay was carried out by the previously described procedure [18]. Briefly, the sample solution was dissolved in DMSO after the solutions were diluted with a phosphate buffer (100 mM KH_2_PO_4_, pH 6.9) become DMSO (10%, *v/v*) concentration. The substrate, *p*-nitrophenyl *α*-*D*-glucoside (Sigma, St. Louis, USA, CAS No. N1377), (*c* 1.5 mM), was prepared by dissolving with phosphate buffer. The tested samples (50 uL) were combined with 100 μL of the *α*-glucosidase enzyme solution (0.35 U/mL, Sigma, St. Louis, MO, USA, CAS. No. G5003), then the mixture was pre-incubated for 10 min at 37 °C. The enzymatic reaction was started by applying substrate (100 μL, *c* 1.5 mM) into the mixture and incubated at 37 °C for 20 min. The reaction was subsequently terminated by the addition of Na_2_CO_3_ (1 mL, *c* 1 M). The absorption was immediately measured at 405 nm by determining the quantity of *p*-nitrophenol released from the substrate. Acarbose was used as a positive control with an IC_50_ value of 0.08 mM.

#### 2.4.2. Nitric Oxide (NO) Production Inhibitory Activity 

The NO inhibitory activity was performed using the same procedure as previously reported [19]. RAW 264.7 (American Type Culture Collection, Manassas, VA, USA) were seeded at 4 × 10^4^ cells/well in 96 well plates suspended in 100 μL DMEM supplemented with 10% FBS and incubated at 37 °C 5% CO_2_ overnight. Cells were incubated with 1 µg/mL LPS for 1 h and treated with various concentrations of sample compounds and vehicle control (DMSO) for 24 h. After 24 h, the NO production inhibitory was determined in the culture supernatant using the Griess reaction by adding 100 µL of Griess reagent in a 96 well plate for 10 min. The determination of nitric oxide was measured at 570 nm with Biochrom EZ Read 400 ELISA microplate reader (Biochrom Ltd., Cambridge, United Kingdom). Additionally, the data were presented as IC_50_ which was calculated with GraphPad Prism 6.0 software. Indomethacin was used as a positive control of NO production inhibitor with an IC_50_ value of 19.61 µM.

#### 2.4.3. Cytotoxicity Assay (MTT Assay)

Cell viability studies were evaluated by the MTT (3-[4,5-dimethylthiazol-2-yl]-2,5-diphenyl tetrazolium bromide) assay [20]. In brief, RAW 264.7 cells (5 × 10^4^ cells/well) were cultured and incubated in 96-well plates for 24 h. The samples were prepared in DMSO at different concentrations (0.01–1000 g/mL). The cells were then treated with samples in DMEM medium (10% FBS, 100 μg/L streptomycin, and 100 IU/mL penicillin at 37 °C in a 5% CO_2_) for 24 h. The medium was then removed and each well was filled with fresh DMEM containing 0.5 mg/mL MTT solution for 4 h at 37 °C in a 5% CO_2_. After that, the medium was removed, and the formazan precipitate was dissolved in DMSO [21]. The absorbance was measured at 550 nm on a microplate reader. All processes were performed in triplicate.

## 3. Results

### 3.1. Isolated Compounds from Broth Extract of D. eschscholtzii SDBR-CMUNKC745

**Figure 3:**
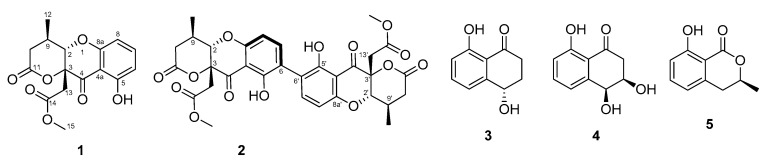
Isolated compounds from *D. eschscholtzii* SDBR-CMUNKC745 broth extract.

The broth extract of *D. eschscholtzii* SDBR-CMUNKC745 (Figure 1) was subjected to various column chromatographic techniques over silica gel or Sephadex LH-20 to afford two new chromone derivatives, daldiniaeschsones A and B (**1** and **2**), together with three known compounds (**3**–**5**) (Figure 3). The three known compounds were identified as (+)-regiolone (isosclerone) (**3**) [22], (+)-3,4-dihydro-3,4,8-trihydroxy-1(2*H*)-naphthalenone (**4**) [23], and (+)-mellein (**5**) [24] by comparing their spectroscopic data with reported data.

Daldiniaeschsone A (**1**), [α]^21^_D_ −1.3 (*c* 1, MeOH), was isolated as a colourless amorphous solid. The molecular formula C_16_H_16_O_7_ was determined from the ^13^C NMR spectroscopic data and HRESITOFMS, which showed an ion peak at *m/z* 321.0972 [M + H]^+^ (calcd for C_16_H_17_O_7_, 321.0969). The IR spectrum showed the characteristic bands for hydroxy (3590 cm^−1^) and carbonyl (1788, 1760, and 1651 cm^−1^) functionalities, while the UV spectrum showed maxima absorption bands at λ_max_ 209, 272, and 354 nm, indicating the presence of a conjugated system in compound **1** (Supplementary Materials, Figures S8 and S9). The ^13^C NMR and DEPT spectroscopic data (Table 1) displayed 16 resonances, including those for two methyls (*δ*_C_ 54.0 and 15.2), two methylenes (*δ*_C_ 40.2 and 37.1), five methines (*δ*_C_ 139.1, 110.5, 108.0, 83.1, 33.9), seven quaternary carbons (*δ*_C_ 194.3 (conjugated ketone carbonyl), 175.3 (ester carbonyl), 169.5 (ester carbonyl), 159.3, 162.3, 107.9, and 84.8). The ^1^H NMR spectroscopic data (Table 1) displayed resonances for the presence of a 1,2,3-trisubstituted benzene ring [*δ*_H_ 7.42 (1H, t, *J* = 8.3 Hz, H-7), 6.56 (1H, dd, *J* = 8.3, 0.9 Hz, H-6), and 6.54 (1H, dd, *J* = 8.3, 0.9 Hz, H-8)], one H-bonded hydroxy proton [*δ*_H_ 11.44 (1H, s, OH-5)], two diastereotopic methylene groups [*δ*_H_ 3.25 (1H, d, *J* = 17.3 Hz, H-13a), 3.18 (1H, d, *J* = 17.3 Hz, H-13b), 2.70 (1H, dd, *J* = 17.3, 8.3 Hz, H-10a) and 2.47 (dd, *J* = 17.3, 8.0 Hz, H-10b)], two methine protons [*δ*_H_ 4.79 (1H, d, *J* = 7.5 Hz, H-2) and 2.96 (1H, hept, *J* = 7.5 Hz, H-9)], and two methyls [*δ*_H_ 3.73 (3H, s, −CO_2_*Me*-15) and 1.32 (3H, d, *J* = 7.5 Hz, H_3_-12)].

Daldiniaeschsone B (**2**), [α]^21^_D_ −3.9 (*c* 1, MeOH), was isolated as a yellow amorphous solid. The molecular formula C_32_H_30_O_14_ was determined from the ^13^C NMR spectroscopic data and HRESITOFMS, which showed an ion peak at *m/z* 661.1504 [M + Na]^+^ (calcd for 661.1528, C_32_H_30_NaO_14_). The IR spectrum showed the characteristic bands for hydroxy (3594 cm^−1^) and carbonyl (1790, 1760, and 1643 cm^−1^) functionalities. The UV spectrum showed maxima absorption bands at λ_max_ 204, 261, and 368 nm. The ^13^C NMR and DEPT spectroscopic data (Table 1) displayed 16 resonances, including those for two methyls (*δ*_C_ 53.8 and 14.9), two methylenes (*δ*_C_ 39.8 and 36.7), four methines (*δ*_C_ 141.2, 107.4, 82.6, 33.5), eight quaternary carbons (*δ*_C_ 194.1 (conjugated ketone carbonyl), 174.9 (ester carbonyl), 169.1 (ester carbonyl), 159.2, 158.4, 117.7, 107.5, and 84.5). The ^1^H NMR spectroscopic data (Table 1) displayed resonances for the presence of a 1,2,3,4-tetrasubstituted benzene ring [*δ*_H_ 7.52 (1H, d, *J* = 8.5 Hz, H-7 and H-7′) and 6.63 (1H, d, *J* = 8.5 Hz, H-8 and H-8′)], one H-bonded hydroxy proton [*δ*_H_ 11.91 (1H, s, OH-5 and OH-5′)], two diastereotopic methylene groups [*δ*_H_ 3.27 (1H, d, *J* = 17.3 Hz, H-13a and H-13a′), 3.20 (1H, d, *J* = 17.3 Hz, H-13b and H-13b′), 2.70 (1H, dd, *J* = 17.3, 8.3 Hz, H-10a and H-10a′) and 2.48 (dd, *J* = 17.3, 8.0 Hz, H-10b and H-10b′)], two methine protons [*δ*_H_ 4.81 (1H, d, *J* = 7.4 Hz, H-2 and H-2′) and 2.98 (1H, hept, *J* = 7.5 Hz, H-9 and H-9′)], and two methyls [*δ*_H_ 3.77 (3H, s, −CO_2_*Me*-15 and CO_2_*Me*-15′) and 1.34 (3H, d, *J* = 7.4 Hz, H_3_-12 and H-12′)].

### 3.2. α-Glucosidase and NO Production Inhibitory Activities

The crude extract and all isolated compounds were evaluated for their α-glucosidase and NO production inhibitory activities (Table 2). The crude extract showed α-glucosidase inhibitory activity with an IC_50_ = 5.69 µg/mL, while compounds **1**–**5** also showed α-glucosidase inhibitory activity with IC_50_ values ranging from 0.16 to 0.85 mM. For the NO production inhibitory activity, the crude extract also showed efficient inhibition with an IC_50_ value of 19.85 µg/mL. Unfortunately, compounds **1** and **2** were inactive, without toxicity, against RAW 264.7 macrophage cells at 50 µg/mL.

## 4. Discussion

### 4.1. Structure Elucidation of Two Novel Compounds Isolated from D. eschscholtzii SDBR-CMUNKC745 Broth Extract

**Figure 4:**
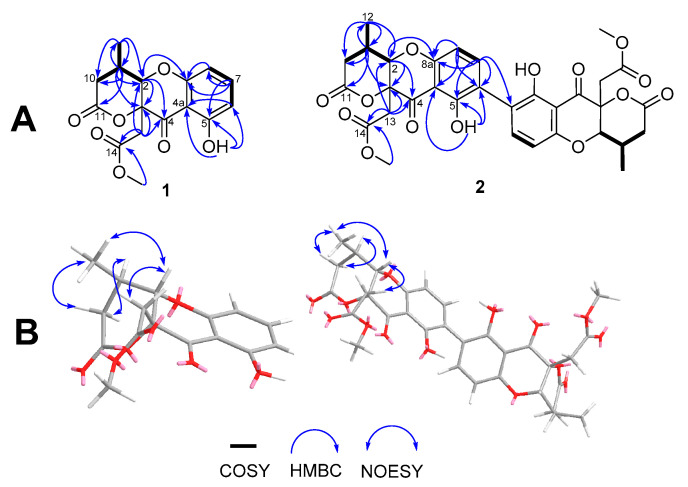
(**A**) Selected HMBC, COSY, and (**B**) NOESY correlations of compounds **1** and **2**.

Analysis of the 2D NMR spectra suggested that daldiniaeschsone A (**1**) had a chromone core structure, which was fused to a *δ*-lactone. The ^1^H-^1^H COSY cross-peaks indicated three coupling systems, CH(6)-CH(7)-CH(8), CH(2)-CH(9)-CH_2_(10) and CH(9)-CH_3_(12) (Figure 4). Additionally, the HMBC correlations from H_3_-12 (*δ*_H_ 1.32) to C-2 (*δ*_C_ 83.1), C-9 (*δ*_C_ 33.9), and C-10 (*δ*_C_ 37.1) revealed the position of the methyl group at C-9. The presence and position of the *δ*-lactone were established on the basis of the HMBC correlations: H-2 (*δ*_H_ 4.79) with C-3 (*δ*_C_ 84.8), C-4 (*δ*_C_ 194.3), C-10, C-12 (*δ*_C_ 15.2), and C-13 (*δ*_C_ 40.2) and H_2_-13 (*δ*_H_ 3.25/3.18) with C-2, C-3, C-4, C-14 (*δ*_C_ 169.5), and H-9 (*δ*_H_ 2.96) with C-3, C-11 (*δ*_C_ 175.3), and C-12. The HMBC correlation of Me-15 (*δ*_H_ 3.73) with C-14 supported the identification of the methyl ester at C-14. While the HMBC correlations of H-7 (*δ*_H_ 7.42) with C-5 (*δ*_C_ 162.3), C-6 (*δ*_C_ 110.5), and C-8a (*δ*_C_ 159.3) and OH-5 (*δ*_H_ 11.44) with C-4a (*δ*_C_ 107.9), C-5, and C-6 indicated that the hydroxy group was attached at C-5. The relative configuration of **1** was evident from the NOESY experiment, as shown in Figure 4. NOESY cross peaks were observed between H-2/H_2_-13, and H-2/H_3_-12, indicating H-2, H_2_-13, and H_3_-12 were located in the same face of the lactone ring. The configuration of the vicinal coupling of H-2 and H-9 were assigned as *trans* based on the magnitude of the coupling constant (*^3^J*) of 7.5 Hz. Thus, the structure of **1** was identified as a daldiniaeschsone A. Compound **1** was subjected to chiral HPLC analysis and the results showed that this compound was a scalemic mixture with an enantiomeric excess (ee) of 15.6% (Supplementary Materials, Figure S19). Due to the small amount of this compound, the separation of its enantiomers was not carried out. 

Daldiniaeschsone B (**2**), [α]^21^_D_ −3.9 (*c* 1, MeOH), was isolated as a yellow amorphous solid, and the molecular formula C_32_H_30_O_14_ was determined based on the HRESITOFMS data (*m/z* 661.1504 [M + Na]^+^) and ^13^C NMR spectroscopic data. The UV and IR spectra were almost identical to those of **1** (Supplementary Materials, Figures S17 and S18). Analysis of 1D and 2D NMR spectroscopic data (Table 1) indicated that **2** shared the same core chromone–lactone structure to **1**. However, the molecular formula C_32_H_30_O_14_ of **2**, along with the observation of only 16 carbon resonances, indicated that this compound was a symmetrical dimer. The ^1^H and ^13^C NMR spectroscopic data of compound **2** were similar to those of **1** except compound **2** displayed ^1^H NMR resonances for an AB aromatic proton system [*δ*_H_/*δ*_C_ 7.52 (1H, d, *J* = 8.5 Hz, H-7/7′)/*δ*_C_ 141.2 and 6.63 (1H, d, *J* = 8.5 Hz, H-8/8′)/*δ*_C_ 107.4] instead of an ABC spin system as in **1**. The HMBC correlations of H-7 with C-5 (*δ*_C_ 159.2), C-6/C-6′ (*δ*_C_ 117.7), and C-8a (*δ*_C_ 158.4) and the lack of resonances for H-6/H-6′ in ^1^H NMR spectral data revealed the C-6/C-6′ carbon–carbon linkage between the two chromone moieties. A detailed assignment of the protons and carbons of **2** is shown in Table 1. Chiral HPLC analysis indicated a scalemic mixture with an ee of 86.2% (Supplementary Materials, Figure S20). Previously, several biphenyl core units have been synthesised by Bringmann et al. [25] and CD spectra have identified their axial/rotameric configuration (a*S* or a*R* isomers). The a*S* isomers of the biphenyls showed Cotton effects (CEs) similar to daldiniaeschsone B, whereas the a*R* isomers showed opposite signs. Shim et al. [26] also reported experimental and calculated CD spectra established a*S*-configuration of three new polyketide biphenyl derivatives, phomalevones A–C, isolated from an unidentified *Phoma*-like fungus. Daldiniaeschsone B displayed a strong negative CE at 225 nm and weak positive CEs at 273 and 317 nm (Supplementary Materials, Figure S21). This was similar to data reported for phomalevones A–C which possess *P*-helicity. Thus, the chirality of the 6,6′-axis of daldiniaeschsone B was assigned as the a*S*-configuration. 

### 4.2. Putative Biosynthesis Pathway of Two Novel Compounds

**Figure 5:**
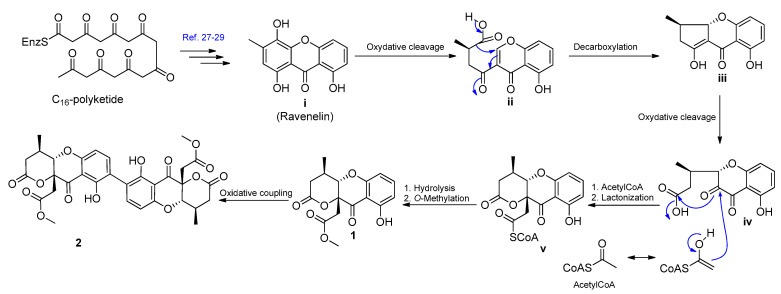
Putative biosynthetic pathway for the polyketides **1** and **2**.

The biosynthetic pathway of monomeric and dimeric polyketide metabolites has been investigated [27]. The putative biosynthetic pathway of compounds **1** and **2** is shown in Figure 5. The multistep condensations of C_16_-octaketide would produce xanthone intermediate **i** (ravenelin) [27,28,29]. Oxidative cleavage of intermediate **i** (ravenelin) could give intermediate **ii**, and further decarboxylation, followed by cyclisation, would give intermediate **iii**. Further oxidative cleavage of intermediate **iii** yields intermediate **iv**. The addition of one acetyl CoA unit will be followed by lactonisation and would give intermediate **v**. Compound **1** could obtain from intermediate **v** via hydrolysis and *O*-methylation, respectively. Finally, the oxidative coupling of compound **1** at C-6/C-6′ would give compound **2**. The scalemic nature of compound **1** would suggest, perhaps, that the oxidative cleavage of **i** (ravenelin) to **ii** is not stereospecific. The significantly higher ee of compound **2** might suggest a kinetic preference (kinetic resolution) for dimerization of one enantiomer of **1**.

### 4.3. α-Glucosidase and NO Production Inhibitory Activities

The broth extract of *D. eschscholtzii* was evaluated for its α-glucosidase inhibitory activity at the concentrations of 100, 150, and 200 µg/mL. At the highest concentration (200 µg/mL), the broth extract showed the highest inhibition of α-glucosidase with the % inhibition of 99.4% and its IC_50_ value was 5.69 µg/mL. However, at the concentrations of 100 and 150 µg/mL, the broth extract had the % inhibition less than 70%, indicating that at these concentrations (100 and 150 µg/mL), the broth extract was inactive. In the case of the pure compounds, compounds **1–5** showed α-glucosidase inhibitory activity with IC_50_ values of 0.16, 0.23, 0.85, 0.55, and 0.76 mM, respectively, while acarbose, the positive control had an IC_50_ = 0.08 mM. The broth extract and the most active compounds (**1** and **2**) were also evaluated for their cytotoxicities against mammalian cells, RAW 264.7 cells, using the MTT assay at the concentrations of 6.25, 12.5, 25, and 50 μg/mL. At the concentration of 50 μg/mL, the broth extract, and compounds **1** and **2** showed cell viability over 75%, indicating that all testing samples had no cytotoxicity at this concentration. The other concentrations (6.25–25 μg/mL) were cytotoxic. Notably, this is the first publication on the α-glucosidase inhibitory activity of these compounds. For the NO production inhibitory activity, the broth extract showed weak activity with an IC_50_ value of 19.85 µg/Ml, whereas compounds **1** and **2** were inactive.

## 5. Conclusions

Previous chemical constituent investigations of *D. eschscholtzii*, using different fungal collections and different conditions of fermentation yielded different compounds. Various types of compounds have been isolated and identified, but polyketides are found as the major compounds. To the best of our knowledge, two new polyketides (chromones) were isolated and identified for the first time in *D. eschscholtzii* isolated from the cinnamon plant (*Cinnamomum bejolghota*) collected from Chiang Mai Province, Thailand. Compounds **1** and **2** are rare tricyclic chromones found in nature, having the chromone unit fused to *δ*-lactone. Compound **1** was a monomer, whereas compound **2** was a symmetrical dimer with a C-6/C-6′ carbon–carbon linkage between the two chromone moieties. The compounds **4** and **5** were discovered from the *Daldinia* genus for the first time. Many chromones isolated from *D. eschscholtzii* have shown good inhibitory activities against *α*-glucosidase and nitric oxide. Unfortunately, in this study, all of the chromone derivatives showed weak *α*-glucosidase inhibitory, and compounds **1** and **2** showed no NO production inhibitory activities.

## Figures and Tables

**Table 1:** ^1^H (500 MHz) and ^13^C (125 MHz) NMR Spectroscopic data of **1** and **2** in CDCl_3._

Position	1		Position	2	
	*δ*c	*δ*_H_ (*J* in Hz)		*δ*c	*δ*_H_ (*J* in Hz)
2	83.1, CH	4.79 (d, 7.5)	2/2′	82.6, CH	4.81 (d, 7.4)
3	84.8, C		3/3′	84.5, C	
4	194.3, C		4/4′	194.1, C	
4a	107.9, C		4a/4a′	107.5, C	
5	162.3, C		5/5′	159.2, C	
6	110.5, CH	6.56 (dd, 8.3, 0.9)	6/6′	117.7, C	
7	139.1, CH	7.42 (t, 8.3)	7/7′	141.2, CH	7.52 (d, 8.5)
8	108.0, CH	6.54 (dd, 8.3, 0.9)	8/8′	107.4, CH	6.63 (d, 8.5)
8a	159.3, C		8a/8a′	158.4, C	
9	33.9, CH	2.96 (hept, 7.5)	9/9′	33.5, CH	2.98 (hept, 7.4)
10a 10b	37.1, CH_2_	2.70 (dd, 17.3, 8.3) 2.47 (dd, 17.3, 8.0)	10a/10a′ 10b/10b′	36.7, CH_2_	2.70 (dd, 17.3, 8.3) 2.48 (dd, 17.3, 8.0)
11	175.3, C		11/11′	174.9, C	
12	15.2, CH_3_	1.32 (d, 7.5)	12/12′	14.9, CH_3_	1.34 (d, 7.4)
13a 13b	40.2, CH_2_	3.25 (d, 17.3) 3.18 (d, 17.3)	13a/13a′ 13b/13b′	39.8, CH_2_	3.27 (d, 17.3) 3.20 (d, 17.3)
14	169.5, C		14/14′	169.1, C	
CO_2_*Me*-15	54.0, CH_3_	3.73 (s)	CO_2_*Me*-15/CO_2_*Me*-15′	53.8, CH_3_	3.77 (s)
OH-5		11.44 (s)	OH-5/OH-5′		11.91 (s)

**Table 2:** α-Glucosidase inhibitory and NO production inhibitory activities of isolated compounds from *D. eschscholtzii* SDBR-CMUNKC745.

Compounds	*α*-Glucosidase Inhibitory Activity		Anti-Inflammatory (NO Production Inhibitory Activity)		% Cell Viability at 50 µg/mL
	% Inhibition at 200 µg/mL	IC_50_ (mM)	% Inhibition at 50 µg/mL	IC_50_ (µM)	
Crude extract	99.4	5.96 ± 0.06 µg/mL	99.5	19.85 ± 0.14 µg/mL	75.52 ± 1.77
**1**	98.7	0.16 ± 0.11	26.5	inactive	88.52 ± 3.97
**2**	96.8	0.23 ± 0.76	36.9	inactive	100.24 ± 1.75
**3**	97.8	0.85 ± 1.19	not tested	not tested	not tested
**4**	97.8	0.55 ± 1.33	not tested	not tested	not tested
**5**	97.5	0.76 ± 0.09	not tested	not tested	not tested
Acarbose	95.6	0.08 ± 0.04	not tested	not tested	not tested
Indomethacin	not tested	not tested	71.0	19.61 ± 0.62	84.96 ± 2.92

## Data Availability

Data are included in the Article/Supplementary Materials/References.

## Supplementary Materials

The following are available online at https://www.mdpi.com/article/10.3390/jof7050358/s1, Figure S1: ^1^H NMR spectrum of compound **1** (500 MHz, CDCl_3_), Figure S2: ^13^C, DEPT90/135 NMR spectra of compound **1** (125 MHz, CDCl_3_), Figure S3: COSY spectrum of compound **1**, Figure S4: HMQC spectrum of compound **1**, Figure S5: HMBC spectrum of compound **1**, Figure S6: NOESY spectrum of compound **1**, Figure S7: HRESITOFMS spectrum of compound **1**, Figure S8: IR (CH_2_Cl_2_) spectrum of compound **1**, Figure S9: UV spectrum of compound **1**, Figure S10: ^1^H NMR spectrum of compound **2** (500 MHz, CDCl_3_), Figure S11: ^13^C and DEPT NMR spectra of compound **2** (125 MHz, CDCl_3_), Figure S12: COSY spectrum of compound **2**, Figure S13: HMQC spectrum of compound **2**, Figure S14: HMBC spectrum of compound **2**, Figure S15: NOESY spectrum of compound **2**, Figure S16: HRESITOFMS spectrum of compound **2**, Figure S17: IR (CH_2_Cl_2_) spectrum of compound **2**, Figure S18: UV spectrum of compound **2**, Figure S19: Chiral HPLC chromatogram of compound **1**, Figure S20: Chiral HPLC chromatogram of compound **2**, Figure S21: ECD spectrum of compound **2**, Figure S22: The isolation and purification of isolated compounds **1–5** from *Daldinia eschscholtzii* SDBR-CMUNKC745 crude extract.
