# Supplementary material for: Daldiniaeschsone A, a Rare Tricyclic Polyketide Having a Chromone Unit Fused to a δ-Lactone and Its Symmetrical Biphenyl Dimer, Daldiniaeschsone B, from an Endophytic Fungus Daldinia eschscholtzii SDBR-CMUNKC745
Source: J Fungi (Basel). 2021 May 2;7(5):358. doi: 10.3390/jof7050358 (PMC8147462; doi:10.3390/jof7050358)
Supplement: Supplementary file 1 [file jof-07-00358-s001.zip › jof-1175927-supplementary.pdf]

Supplementary Material

Daldiniaeschsone A, a rare tricyclic polyketide having a chromone unit fused to a δ-lactone and its symmetrical biphenyl dimer, daldiniaeschsone B, from an endophytic fungus *Daldinia eschscholtzii* SDBR-CMUNKC745

Natnicha Wutthiwong,[1,2] Virayu Suthiphasilp,[3] Aknarin Pintatum,[3] Nakarin Suwannarach,[1,2] Jaturong Kumla,[1,4] Saisamorn Lumyong,[1,2,4*] Tharakorn Maneerat,[3,5] Rawiwan Charoensup,[5,6] Sarot Cheenpracha,[7] Thunwadee Limtharakul,[8] Stephen G. Pyne,[9] Surat Laphookhieo[3,5*]

[1]Department of Biology, Faculty of Science, Chiang Mai University, Chiang Mai 50200, Thailand.

[2]Research Center of Microbial Diversity and Sustainable Utilization, Chiang Mai University, Chiang Mai 50200, Thailand.

[3]Center of Chemical Innovation for Sustainability (CIS), and School of Science, Mae Fah Luang University, Chiang Rai 57100, Thailand

[4]Academy of Science, The Royal Society of Thailand, Bangkok, Thailand

[5]Medicinal Plants Innovation Center of Mae Fah Luang University, Chiang Rai 57100, Thailand

[6]School of Integrative Medicine, Mae Fah Luang University, Chiang Rai 57100, Thailand

[7]School of Science, University of Phayao, Phayao 56000, Thailand

[8]Department of Chemistry, Faculty of Science and Research Center on Chemistry for Development of Health Promoting Products from Northern Resources, Chiang Mai University, Chiang Mai 50200, Thailand

[9]School of Chemistry and Molecular Bioscience, University of Wollongong, Wollongong, New South Wales 2522, Australia

Content **Page**

Figure S1. ^{1}H NMR spectrum of compound **1** (500 MHz, $CDCl_3$) 3

Figure S2. ^{13}C, DEPT90/135 NMR spectra of compound **1** (125 MHz, $CDCl_3$) 3

Figure S3. COSY spectrum of compound **1** 4

Figure S4. HMQC spectrum of compound **1** 4

Figure S5. HMBC spectrum of compound **1** 5

Figure S6. NOESY spectrum of compound **1** 5

Figure S7. HRESITOFMS spectrum of compound **1** 6

Figure S8. IR (CH_2Cl_2) spectrum of compound **1** 6

Figure S9. UV spectrum of compound **1** 7

Figure S10. ^{1}H NMR spectrum of compound **2** (500 MHz, $CDCl_3$) 7

Figure S11. ^{13}C and DEPT NMR spectra of compound **2** (125 MHz, $CDCl_3$) 8

Figure S12. COSY spectrum of compound **2** 8

Figure S13. HMQC spectrum of compound **2** 9

Figure S14. HMBC spectrum of compound **2** 9

Figure S15. NOESY spectrum of compound **2** 10

Figure S16. HRESITOFMS spectrum of compound **2** 10

Figure S17. IR (CH_2Cl_2) spectrum of compound **2** 11

Figure S18. UV spectrum of compound **2** 11

Figure S19. Chiral HPLC chromatogram of compound **1** 12

Figure S20. Chiral HPLC chromatogram of compound **2** 13

Figure S21. ECD spectrum of compound **2** 14

Figure S22. The isolation and purification of isolated compounds **1-5** from *Daldinia eschscholtzii* SDBR-CMUNKC745 crude extract 14

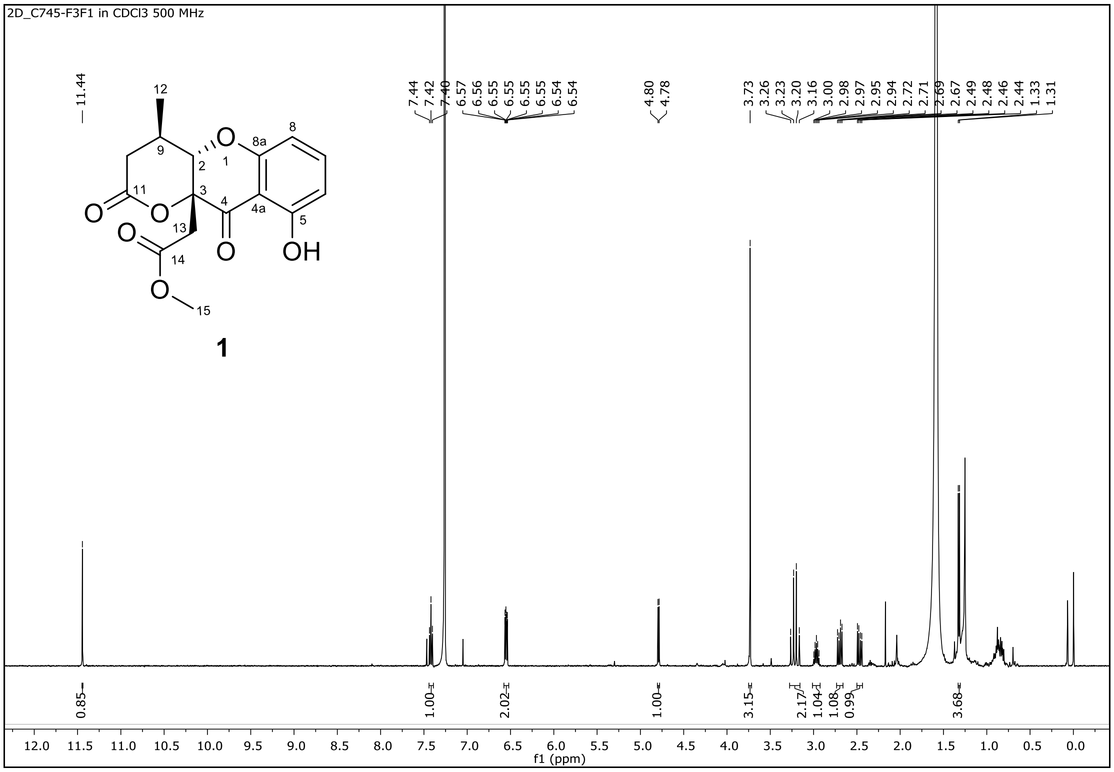

Figure S1. ^{1}H NMR spectrum of compound **1** (500 MHz, $CDCl_3$)

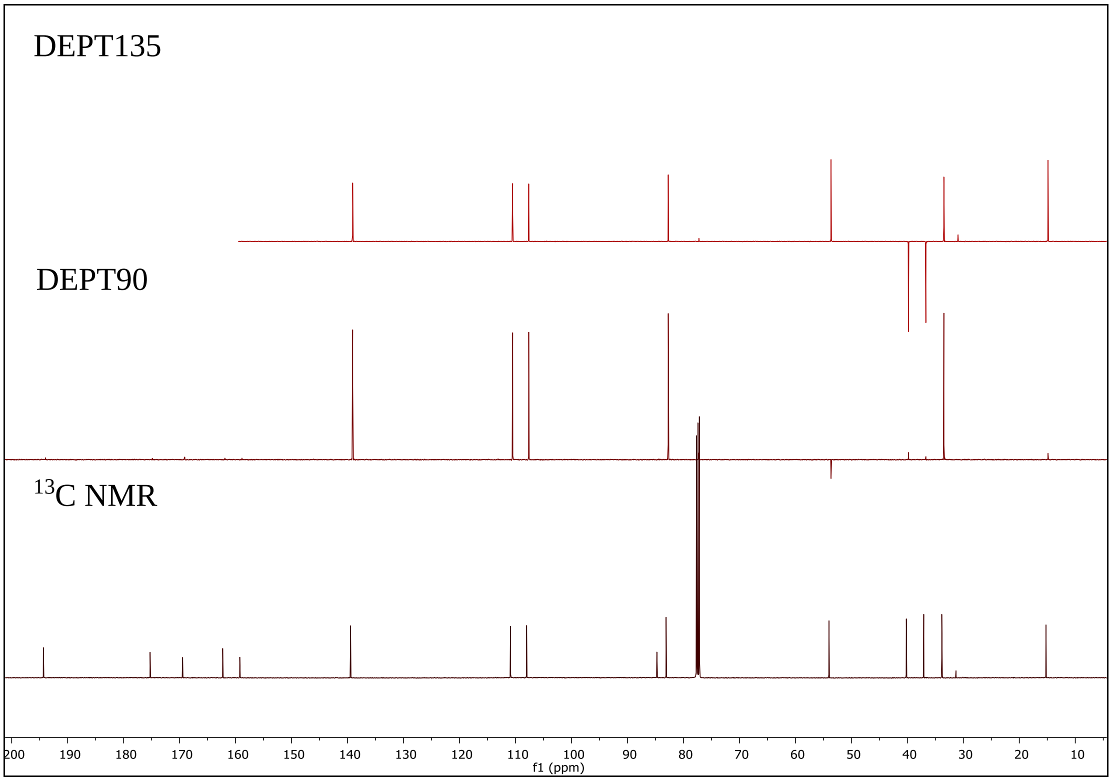

Figure S2. ^{13}C and DEPT NMR spectra of compound **1** (125 MHz, $CDCl_3$)

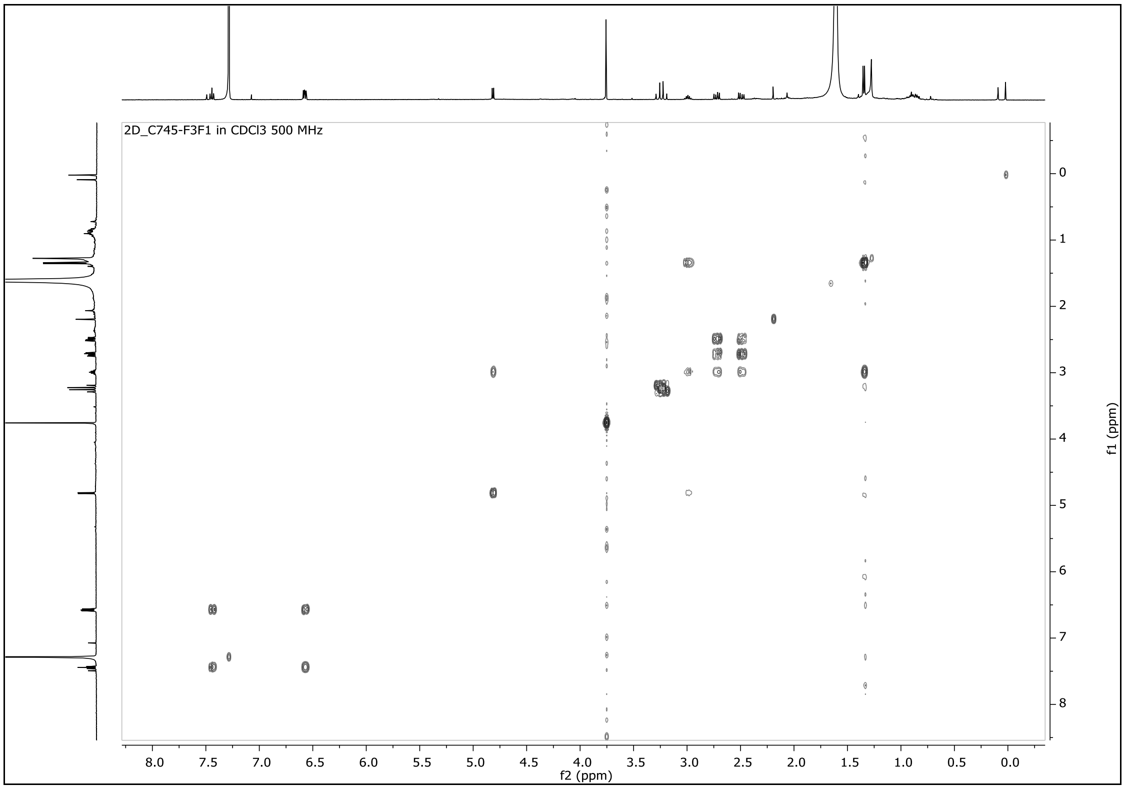

Figure S3. COSY spectrum of compound **1**

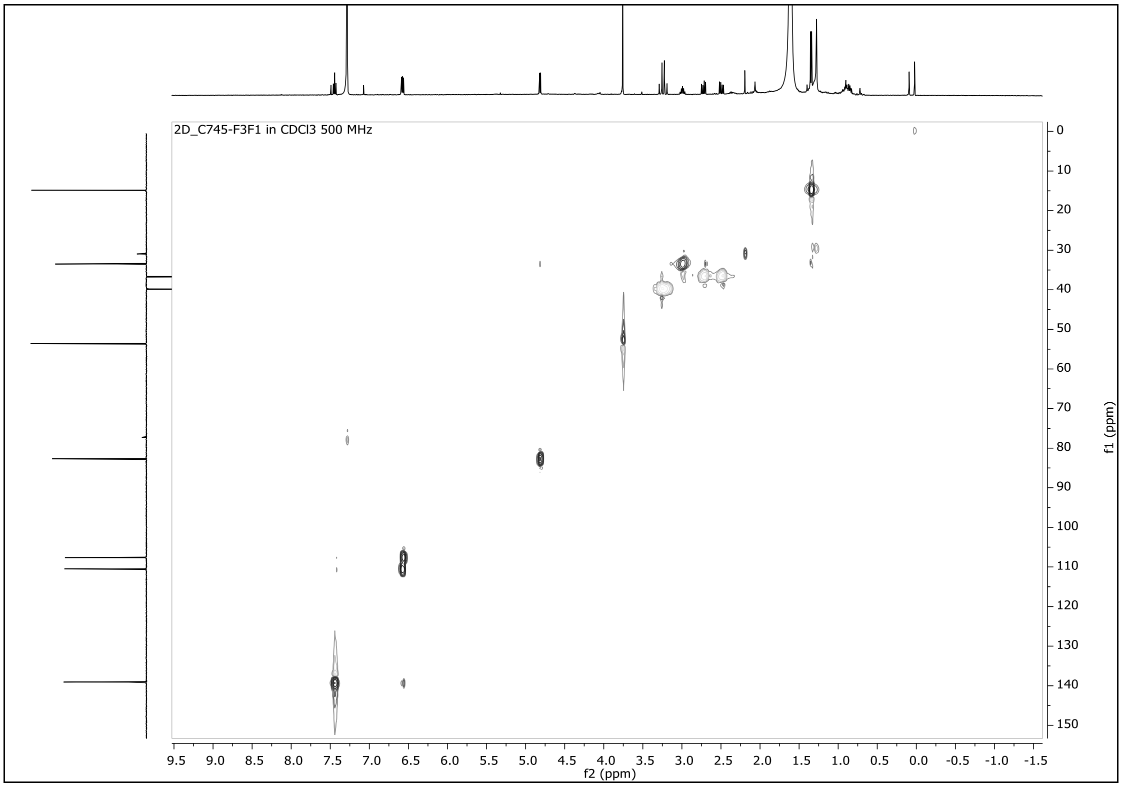

Figure S4. HMQC spectrum of compound **1**

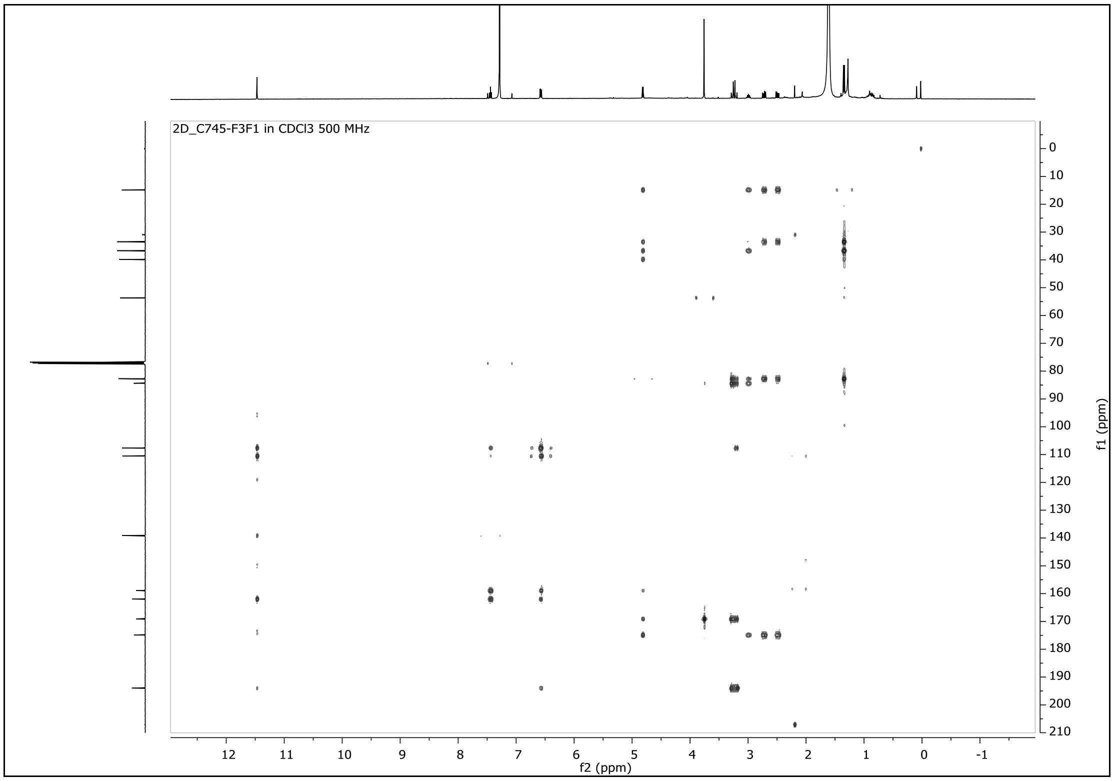

Figure S5. HMBC spectrum of compound **1**

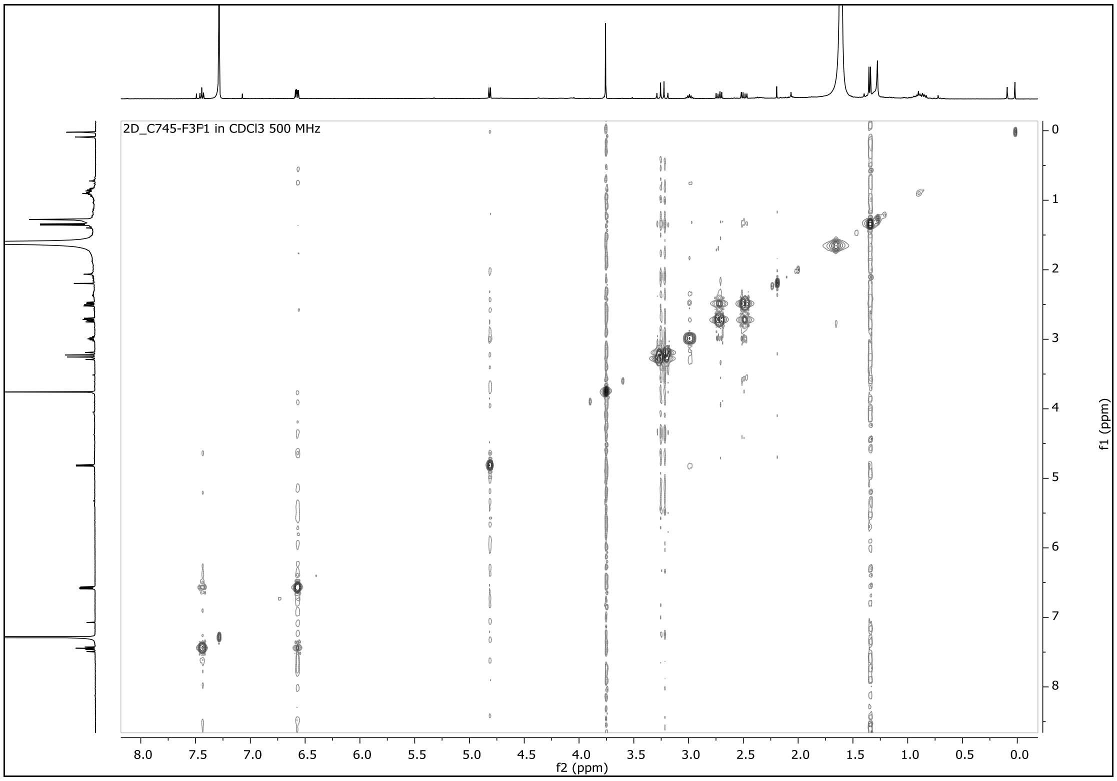

Figure S6. NOESY spectrum of compound **1**

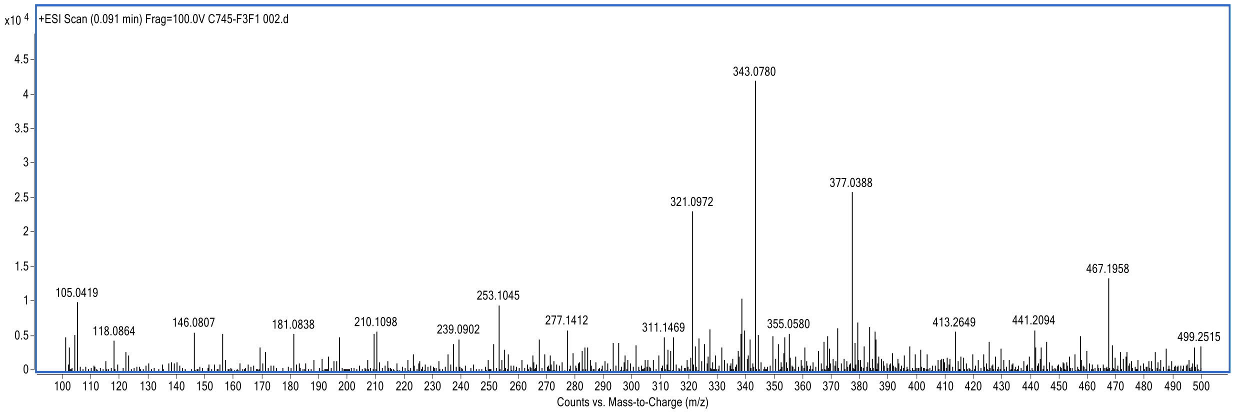

Formula (M)	Score (MFG)	Mass	Mass (MFG)	m/z (Calc)	Diff (ppm)	DBE	m/z
C16 H16 O7	99.66	320.0899	320.0896	321.0969	-1	9	321.0972

Figure S7. HRESITOFMS spectrum of compound **1**

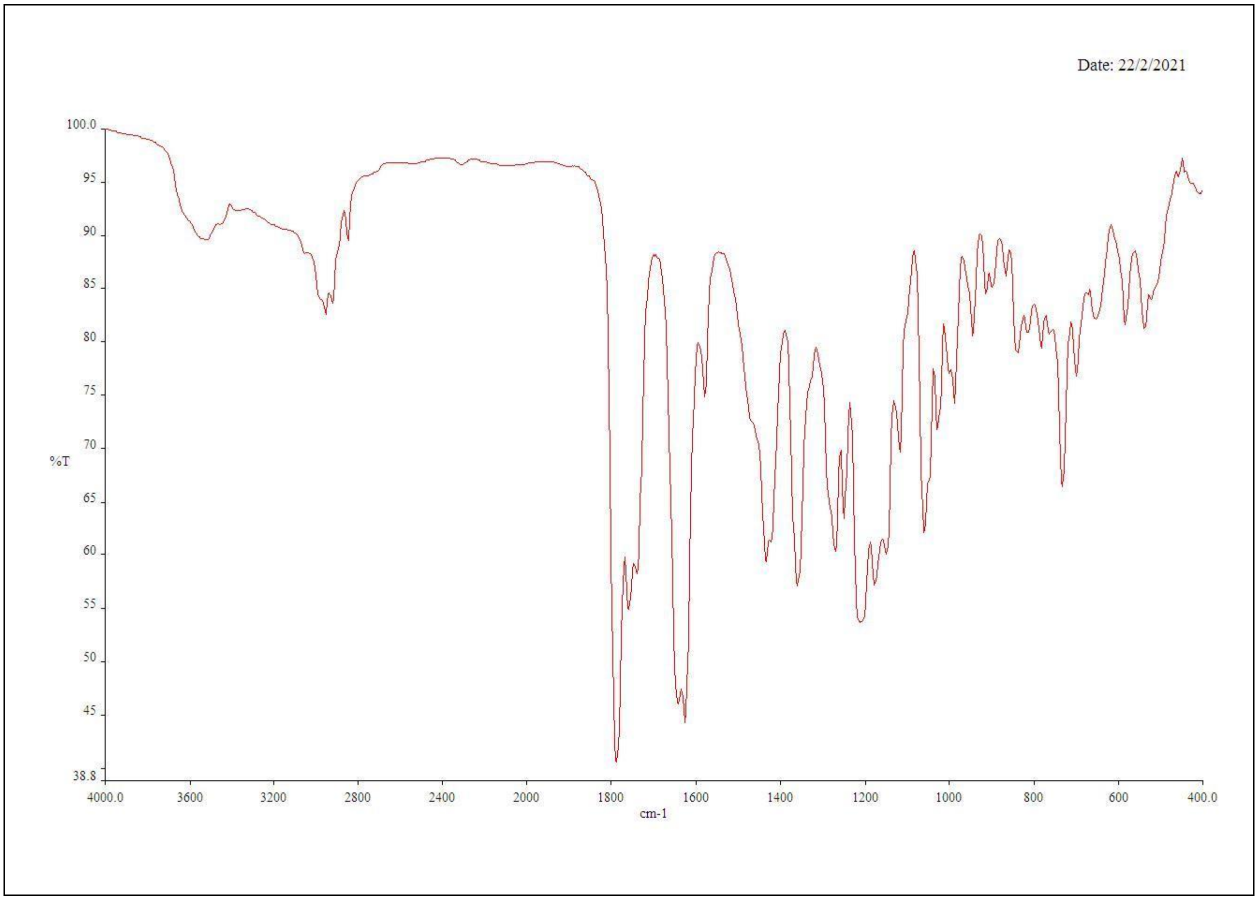

Figure S8. IR (CH_2Cl_2) spectrum of compound **1**

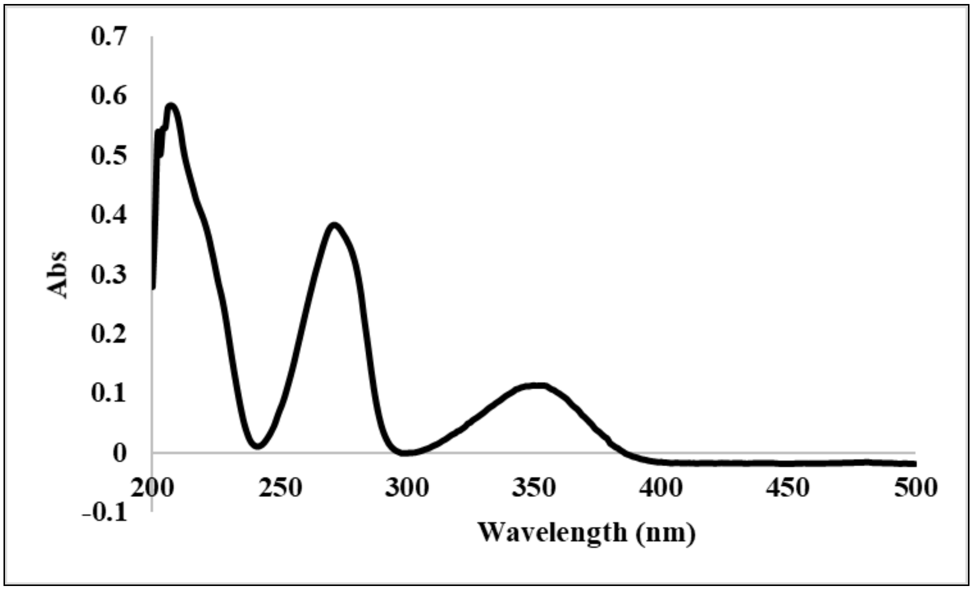

Figure S9. UV spectrum of compound **1**

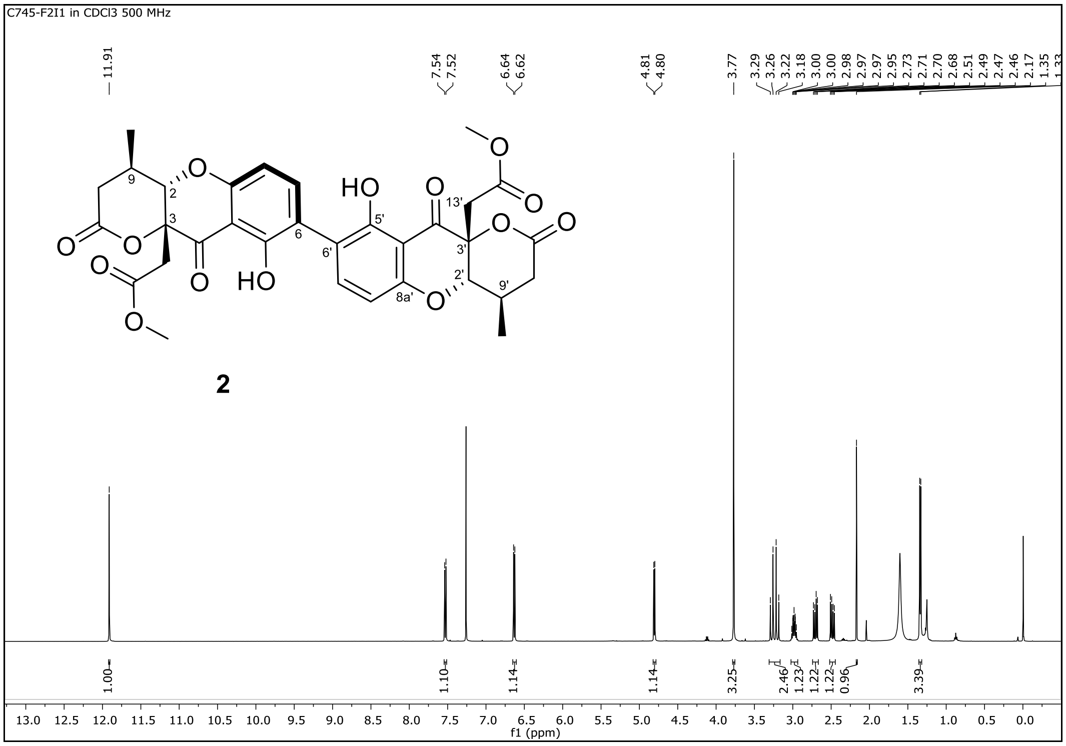

Figure S10. ^{1}H NMR spectrum of compound **2** (500 MHz, $CDCl_3$)

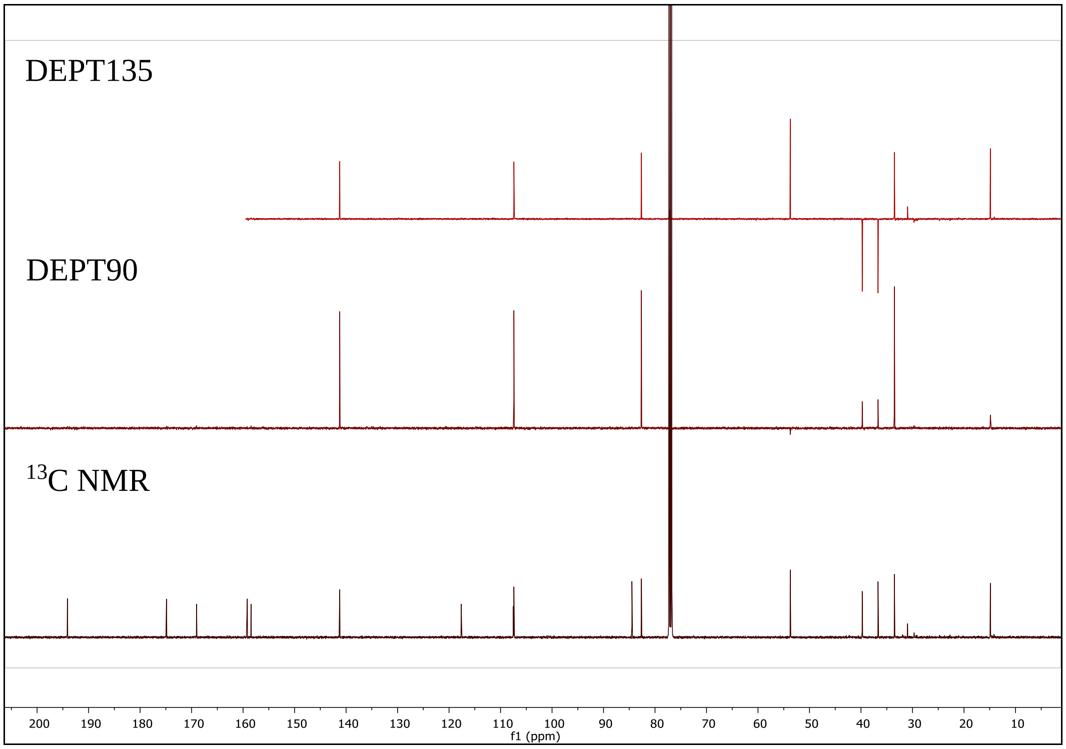

Figure S11. ^{13}C, DEPT90/135 NMR spectra of compound **2** (125 MHz, $CDCl_3$)

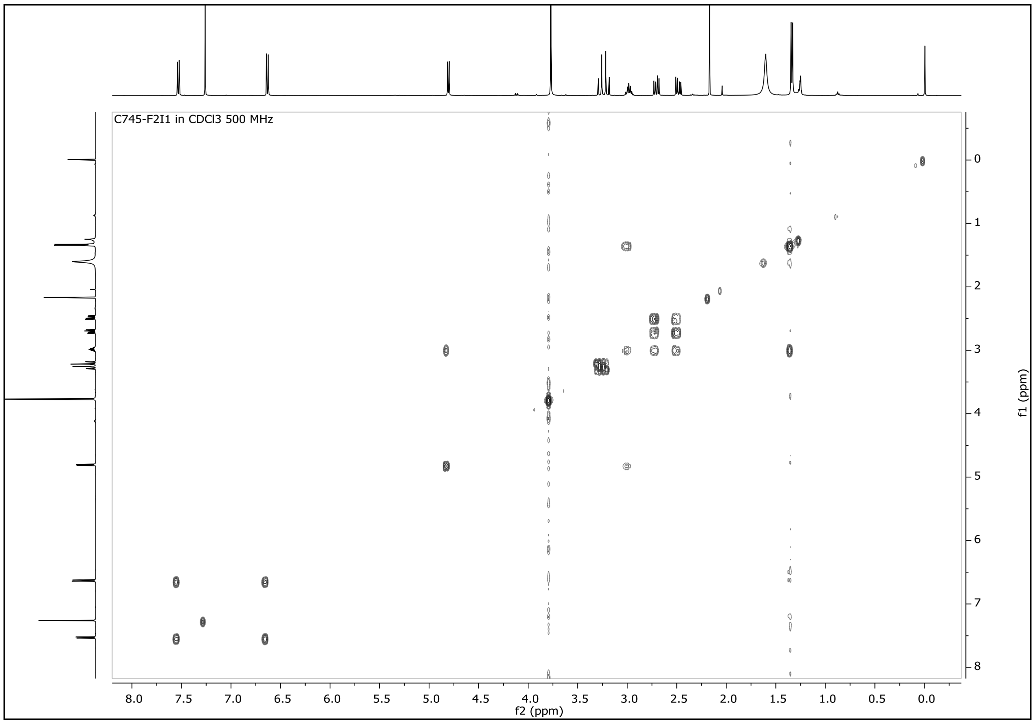

Figure S12. COSY spectrum of compound **2**

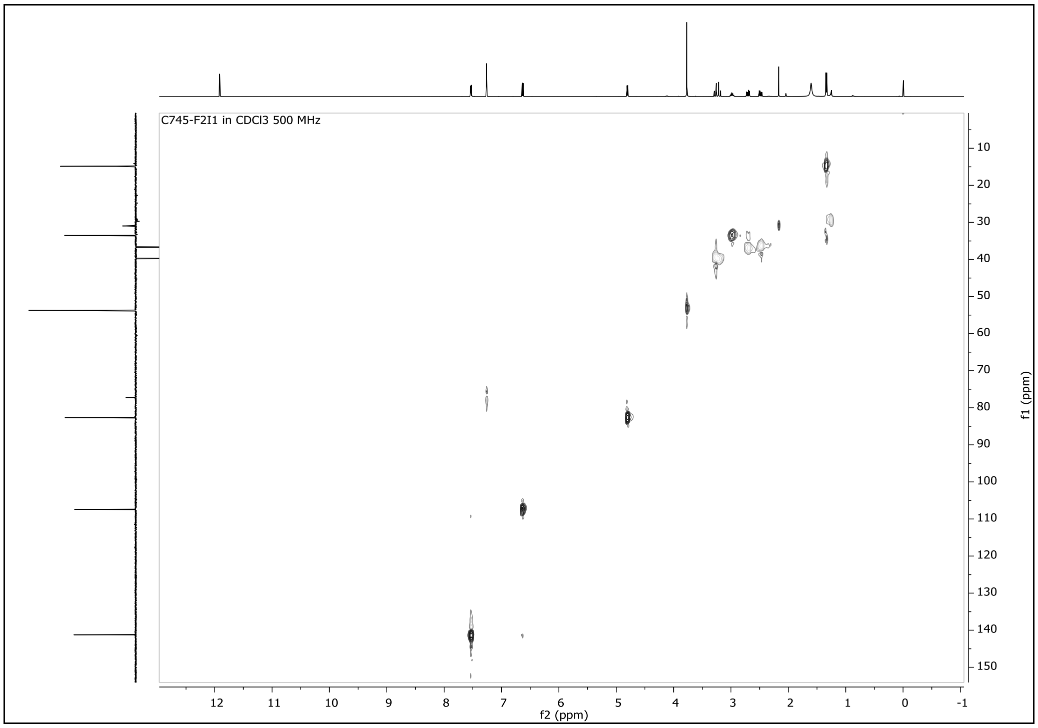

Figure S13. HMQC spectrum of compound **2**

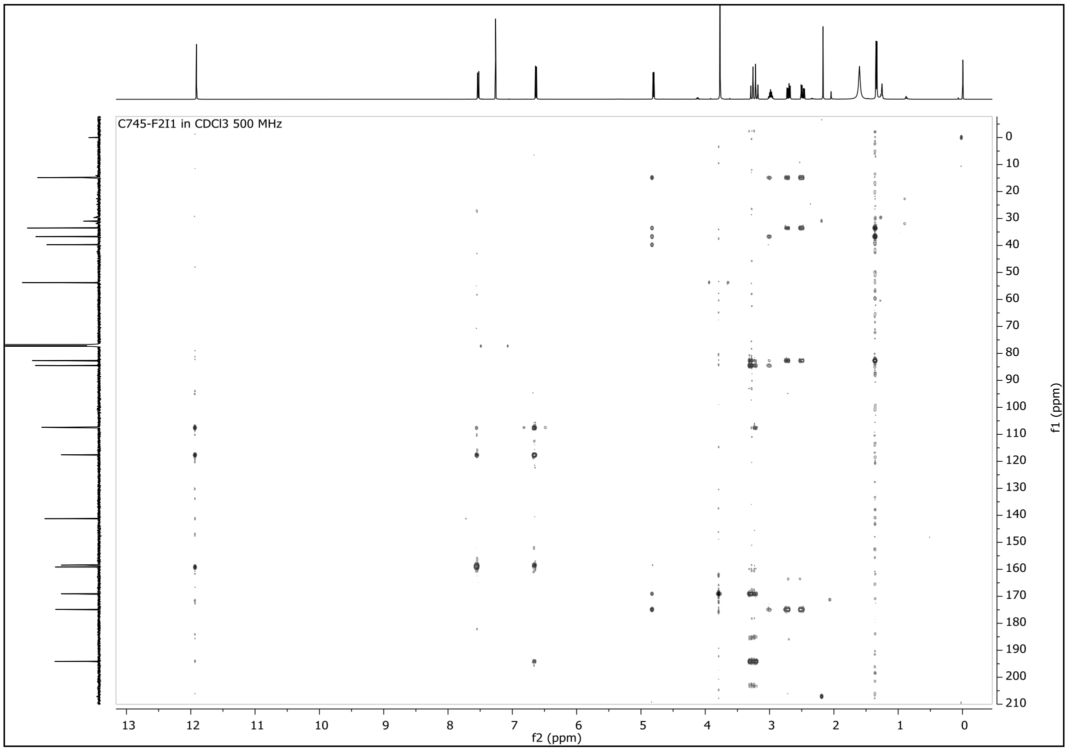

Figure S14. HMBC spectrum of compound **2**

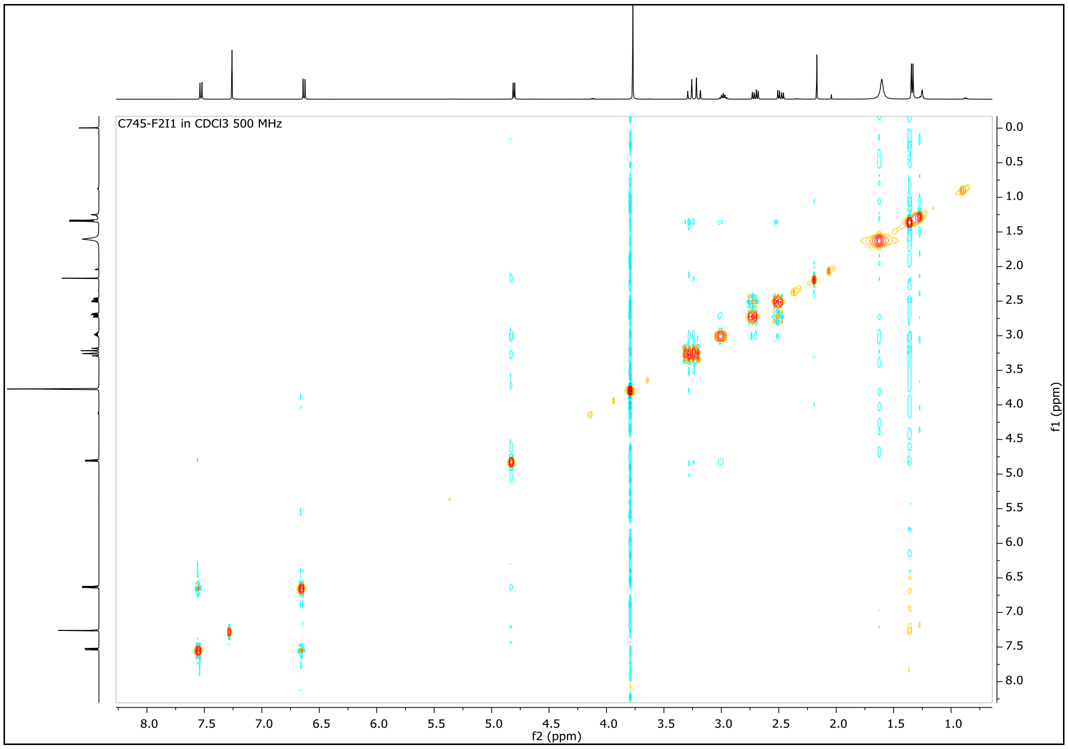

Figure S15. NOESY spectrum of compound **2**

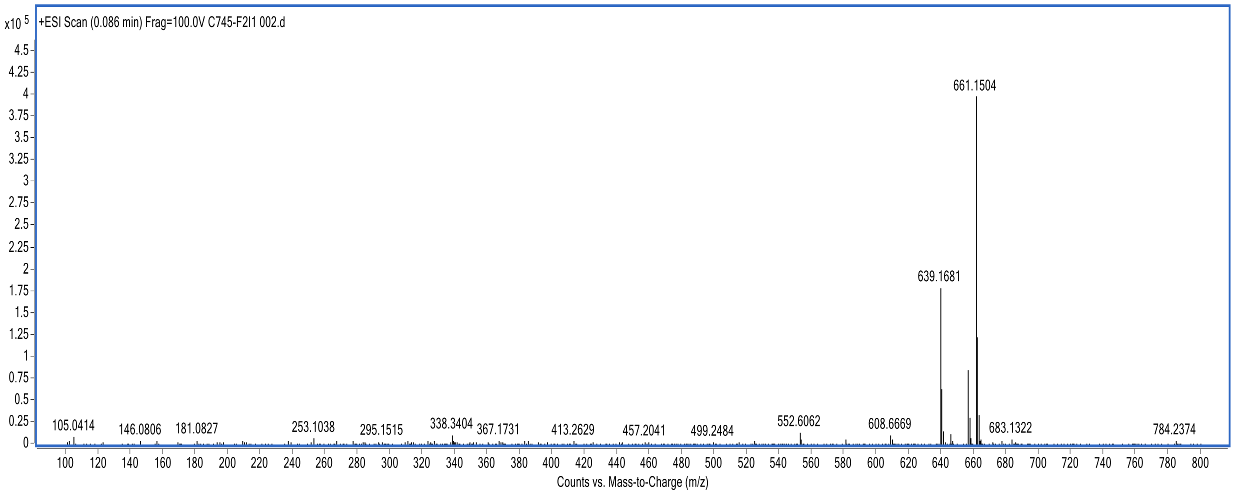

Formula (M)	Score (MFG)	Mass	Mass (MFG)	m/z (Calc)	Diff (ppm)	DBE	m/z
C41 H24 O9	98.28	660.1431	660.142	661.1493	-1.65	30	661.1504
C32 H29 Na O14	92.39	660.1431	660.1455	661.1528	3.6	18	661.1504
Formula (M)	Score (MFG)	Mass	Mass (MFG)	m/z (Calc)	Diff (ppm)	DBE	m/z
C32 H30 O14	89.83	638.1608	638.1636	639.1708	4.28	18	639.1681
C39 H26 O9	87.04	638.1608	638.1577	639.165	-4.92	27	639.1681

Figure S16. HRESITOFMS spectrum of compound **2**

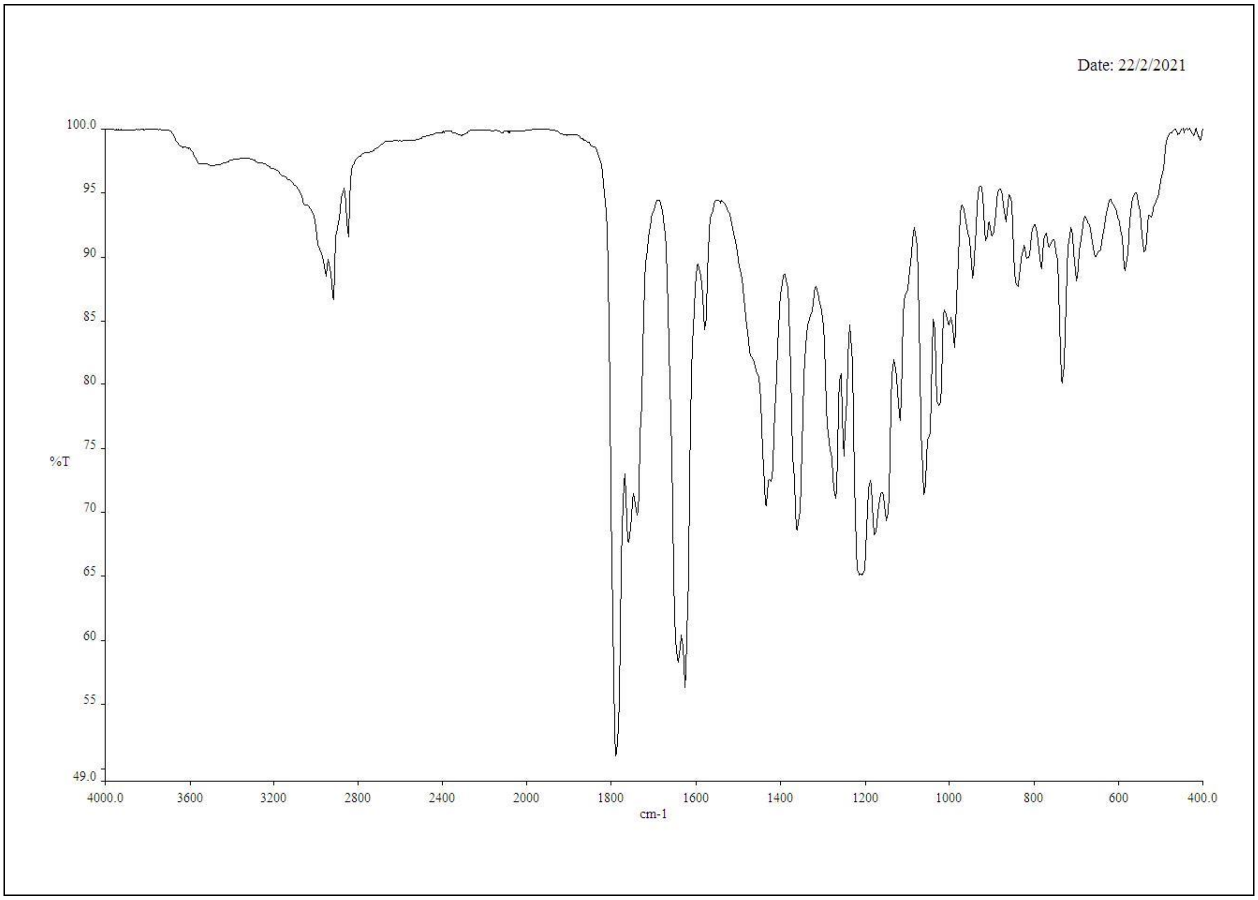

Figure S17. IR (CH_2Cl_2) spectrum of compound **2**

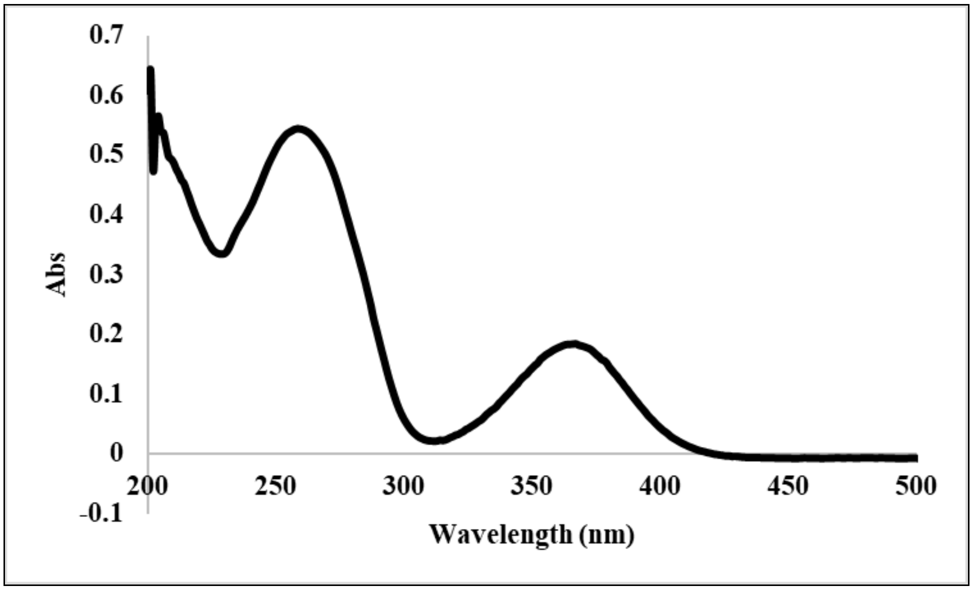

Figure S18. UV spectrum of compound **2**

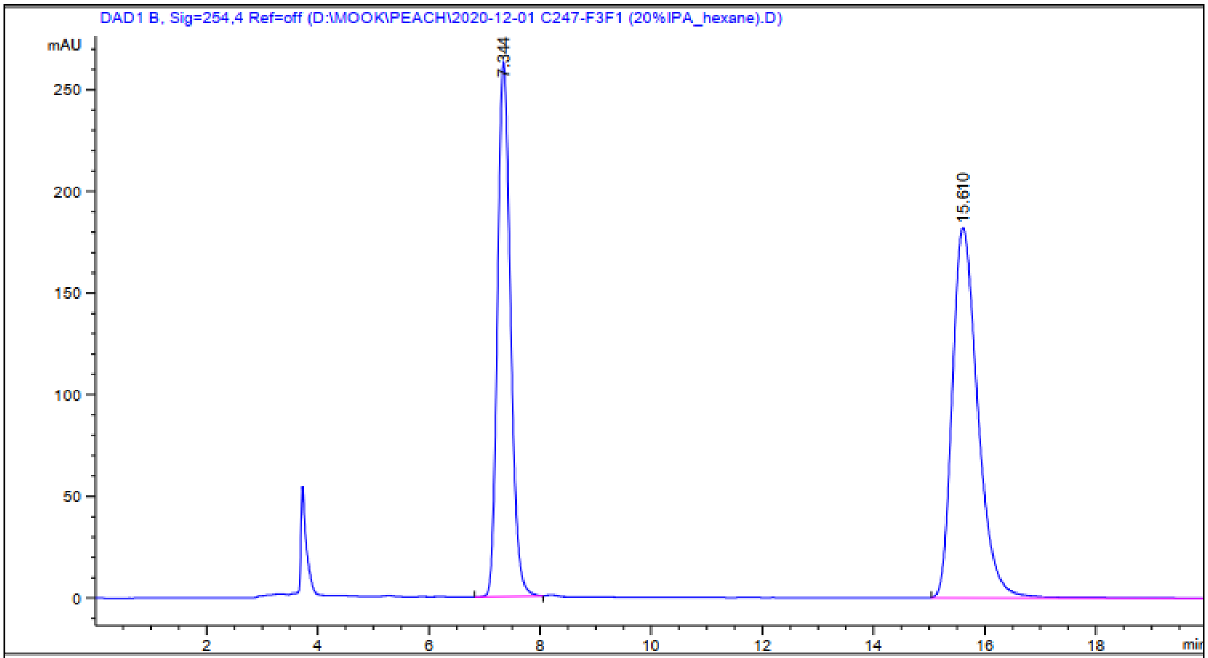

===

Area Percent Report

===

Sorted By : Signal
Multiplier : 1.0000
Dilution : 1.0000
Sample Amount: : 1.00000 [ng/ul] (not used in calc.)
Use Multiplier & Dilution Factor with ISTDs

Signal 1: DAD1 B, Sig=254,4 Ref=off

Peak #	RetTime [min]	Type	Width [min]	Area [mAU*s]	Height [mAU]	Area %
1	7.344	BB	0.2503	4242.00586	262.81641	42.1957
2	15.610	HBA	0.4898	5811.15918	182.35333	57.8043

Figure S19. Chiral HPLC chromatogram of compound **1**

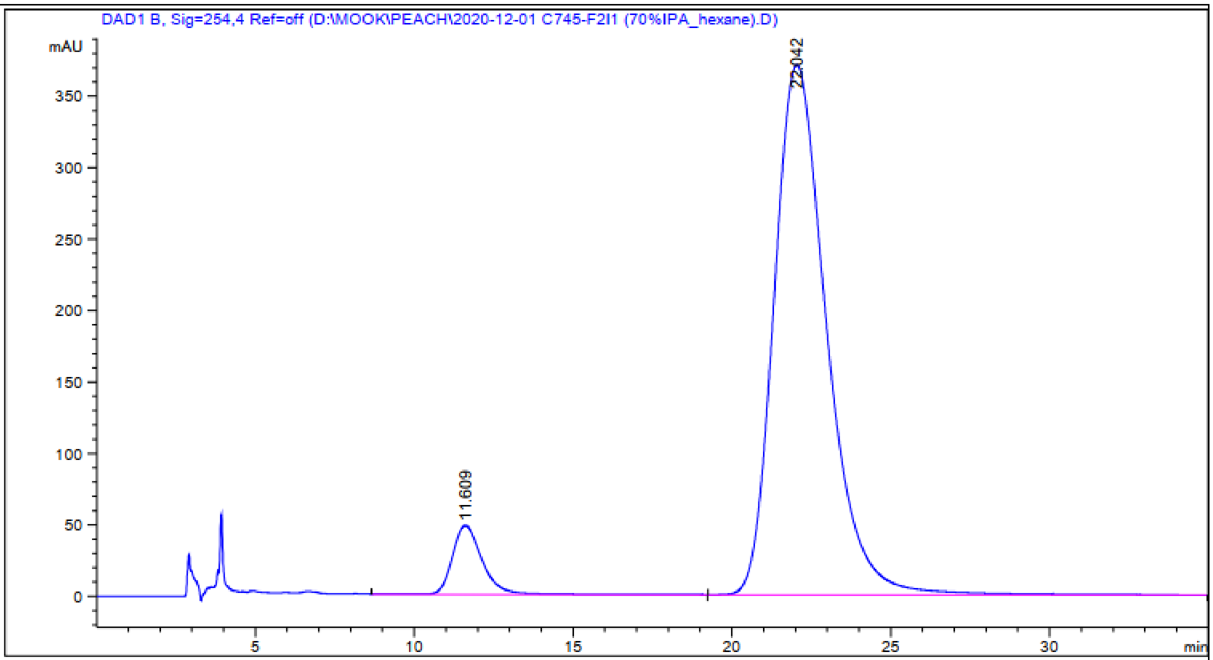


```
======================================================================
                        Area Percent Report
======================================================================

Sorted By             :      Signal
Multiplier            :      1.0000
Dilution              :      1.0000
Sample Amount:               :      1.00000  [ng/ul]   (not used in calc.)
Use Multiplier & Dilution Factor with ISTDs


Signal 1: DAD1 B, Sig=254,4 Ref=off

Peak RetTime Type  Width     Area      Height     Area
  #   [min]        [min]   [mAU*s]     [mAU]        %
----|-------|----|-------|----------|----------|--------|
   1  11.609 VB R  0.9827 3149.54663   48.18493    6.8926
   2  22.042 BBA   1.7622 4.25452e4   370.92563   93.1074
```

Figure S20. Chiral HPLC chromatogram of compound **2**

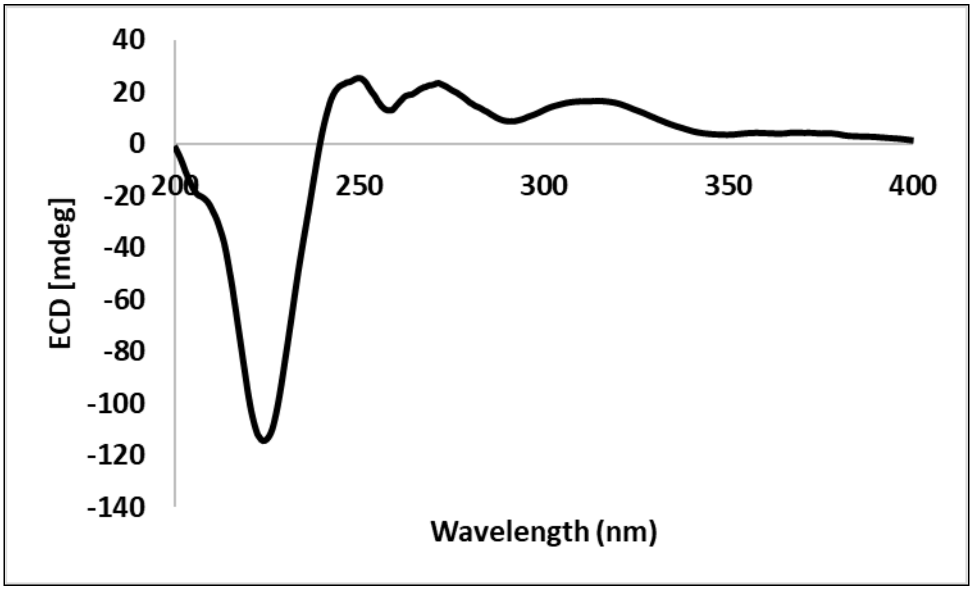

Figure S21. ECD spectrum of compound **2**

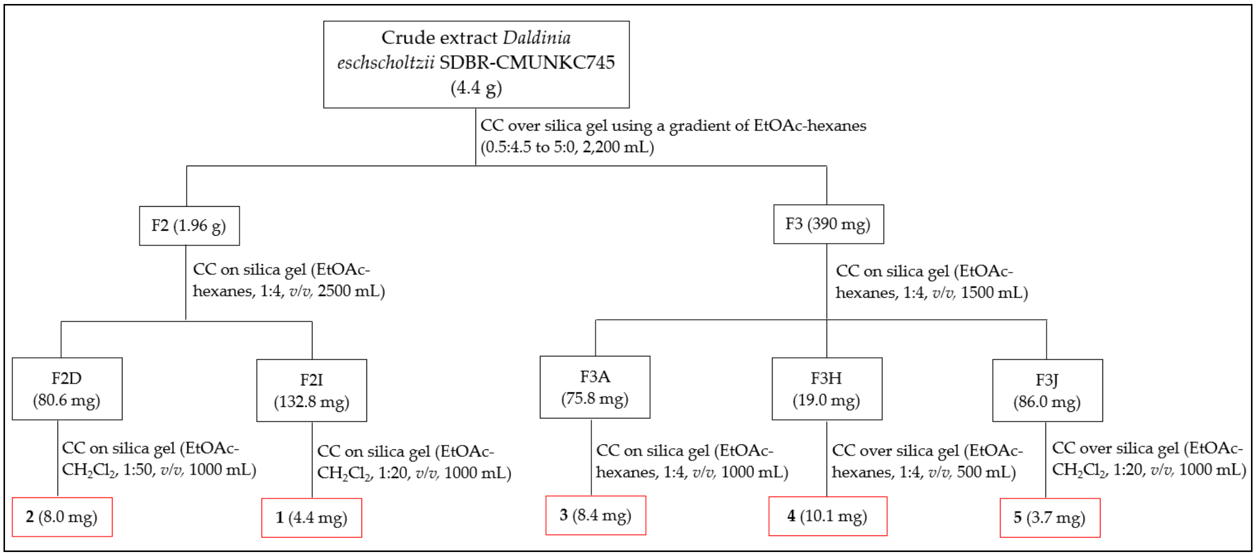

Figure S22. The isolation and purification of isolated compounds **1-5** from *Daldinia eschscholtzii* SDBR-CMUNKC745 crude extract
